# Motion-correction strategies for enhancing whole-body PET imaging

**DOI:** 10.3389/fnume.2024.1257880

**Published:** 2024-02-07

**Authors:** James Wang, Dalton Bermudez, Weijie Chen, Divya Durgavarjhula, Caitlin Randell, Meltem Uyanik, Alan McMillan

**Affiliations:** 1 Department of Radiology, University of Wisconsin Madison, Madison, WI, United States,; 2 Department of Medical Physics, University of Wisconsin Madison, Madison, WI, United States,; 3 Department of Electrical and Computer Engineering, University of Wisconsin Madison, Madison, WI, United States,; 4 Department of Computer Science, University of Wisconsin Madison, Madison, WI, United States,; 5 Department of Biomedical Engineering, University of Wisconsin Madison, Madison, WI, United States,; 6 Data Science Institute, University of Wisconsin Madison, Madison, WI, United States

**Keywords:** positron emission tomography (PET), respiratory gating, cardiac gating, motion correction, hardware-driven gating, data-driven gating

## Abstract

Positron Emission Tomography (PET) is a powerful medical imaging technique widely used for detection and monitoring of disease. However, PET imaging can be adversely affected by patient motion, leading to degraded image quality and diagnostic capability. Hence, motion gating schemes have been developed to monitor various motion sources including head motion, respiratory motion, and cardiac motion. The approaches for these techniques have commonly come in the form of hardware-driven gating and data-driven gating, where the distinguishing aspect is the use of external hardware to make motion measurements vs. deriving these measures from the data itself. The implementation of these techniques helps correct for motion artifacts and improves tracer uptake measurements. With the great impact that these methods have on the diagnostic and quantitative quality of PET images, much research has been performed in this area, and this paper outlines the various approaches that have been developed as applied to whole-body PET imaging.

## 1 Introduction

Positron emission tomography (PET) imaging has been widely used medically, with its most predominant use in oncologic imaging (1). Here, PET can be used as a tool for whole-body examinations to assess cancer growth or spread. Additionally, PET imaging has clinical applications in cardiology, neurology, and psychiatry (2). However, an unavoidable issue in PET imaging stems from its time-resolved nature of acquisition and thus its inherent sensitivity to subject motion occurring during image acquisition. Image artifacts due to motion can significantly degrade image quality and compromise diagnostic accuracy. Motion can stem from a multitude of physiological sources including respiration, cardiac motion, and bulk patient motion.

Motion can cause both emission-emission misalignment and transmission-emission misalignment in PET imaging (3). A transmission scan is typically acquired for attenuation correction, but the introduction of motion can cause a misalignment between transmission and emission scans, leading to incorrect attenuation correction factors being applied. Emission-emission misalignments stem from motion occurring either within or between emission scans which can lead to additional complications such as errors in physiological parameters. Getting accurate values is especially important when it comes to quantitative kinetic modeling. The presence of motion-induced blurring can lead to errors in ROI definition. Tracer distribution changes over time, and motion can disrupt the natural flow, thereby affecting the reliability of kinetic modeling. When incorrect physiological parameters are used in the kinetic modeling process, such as in the Patlak analysis, it can lead to inaccurate and unreliable results; changes to kinetic modeling parameters such as distribution volume ratio or metabolic flux can significantly impact the reliability of the analysis (3, 4). Hence, motion correction is a crucial step for PET to improve the quality and accuracy of the obtained images, especially with the development of long axial field of view PET scanners with near or complete total body coverage.

Various techniques have been employed and proposed to minimize the effects of different sources of motion (5). Subject motion can be categorized as either voluntary or involuntary. One technique that is used to restrict voluntary motion is the use of physical constraints, a commonly used approach across multiple imaging modalities that constricts the movement of subjects with physical padding and straps (6). However, this method alone can be inadequate in keeping a subject from inadvertently making small involuntary movements that can still adversely affect the image quality. The severity of motion artifacts is also dependent on the scanner that is being used. Whole-body scanners are able to achieve shorter scan times and thereby reduce motion blurring (7). This would mainly impact bulk motion and respiratory motion (typically 12–18 breaths per minute) and not cardiac motion (typically 60–100 beats per minute), since the cardiac rate is still much higher than the acquisition rate. Furthermore, multiple types of motion can occur simultaneously within the single field of view (e.g., bulk head motion and cardiac motion), which may require multiple, simultaneous gating schemes.

As such, one of the essential techniques employed in PET imaging is gating, which helps further minimize motion artifacts caused by involuntary patient movement (5, 8). This technique allows patients to breathe freely and increase comfort and accessibility (9). Furthermore, this technique can be applied in conjunction with physical constraints to help improve image quality. Two main approaches have been explored to address this challenge: hardware-driven gating and data-driven gating. Hardware-driven gating uses sensors distinct from the PET imaging system. The signals acquired from these devices are what allow for prospective and retrospective motion correction across medical imaging devices such as MRI and CT. The prospective approach is achieved by synchronizing image acquisition with the patient’s measured physiological signals, such as respiration or cardiac activity, to reconstruct images at specific phases or amplitudes in the respective motion cycles. Although prospective gating is possible for PET imaging, retrospective gating is most commonly utilized due to its greater flexibility in modern PET scanners that utilize list-mode acquisition (10). Hardware-driven retrospective gating approaches are achieved by combining externally measured signals with the raw PET data after acquisition. On the other hand, data-driven gating retrospectively analyzes the raw PET data to extract motion information rather than obtaining this information from an external device. Each of these approaches has its advantages and limitations, especially when considering specific regions of the body being imaged. By combining these approaches, motion correction in PET imaging can be optimized to achieve superior quantitative and qualitative images and relevant measures.

### 1.1 Technical foundations and clinical applications of PET imaging

PET imaging targets a wide range of molecular and physiological processes, providing valuable diagnostic and prognostic information in various clinical applications. However, there are fundamental limits that come with PET imaging that impact the spatial resolution of acquired images. There are three main factors that affect PET resolution: detector geometry, annihilation photon acollinearity, and positron range (11).

PET scanners operate by having an array of detectors in a ring to capture the emitted annihilation photons resulting from positron emitting radioactive tracers. Each element of the detectors used in the ring must be large enough so that it can be sensitive enough to detect the incoming gamma rays. Having smaller detectors could provide better spatial resolution and help minimize parallax error, but it would come with the fundamental trade-off of noise (12). Acollinearity is also an inherent issue in PET imaging that arises from the electron-positron annihilation process not being emitted at exactly 180° (1). With the line of responses not intersecting at the right location, this can introduce blurring and distortions. However, these issues are relatively fixed per system and can be minimized by using a small-ring detector setup (11). The positron annihilation range is an inherent uncertainty that stems from positrons having their annihilation events at a distance away from the source. This leads to a spatial blurring effect that scales with higher positron energies that can travel further before undergoing annihilation.

As such, the radionuclide used as a tracer for PET imaging not only has a direct impact on the biological location of radiotracer uptake but also on the image resolution. Hence, there are a wide variety of radiotracers available that can be chosen depending on the specific clinical application and the biological process being investigated. Fluorine-18 is the most commonly used isotope in PET scans and is used extensively in oncology as ^18^F-FDG (fluorodeoxyglucose) (13). This isotope not only has a long half-life of approximately 110 min, but also emits low-energy positrons which effectively create a positron range (FWHM) of 0.54 mm (13, 14). With a small positron range, ^18^F can provide high image resolution. These isotopes can be used in different compounds as PET radiotracers such as FDG, NaF, FDDNP, and FDOPA. FDG is widely used in oncology since it is a glucose analog and able to be used to detect increased glucose metabolism by cancer cells. Although ^18^F is an ideal radionuclide for PET imaging, there is a codependence when developing radiotracers, so other isotopes have also been explored such as ^11^C, ^89^Zr, ^124^I, ^68^Fa, and ^90^Y (15). The initial development of radiotracers looked at elements commonly found in the human body, which resulted in ^15^O, ^13^N, and ^11^C being used as isotopes. Novel PET radiotracers continue to be developed using a variety of radioisotopes for an ever-growing number of applications.

### 1.2 Commonly imaged body regions and motion

**FIGURE 1:**
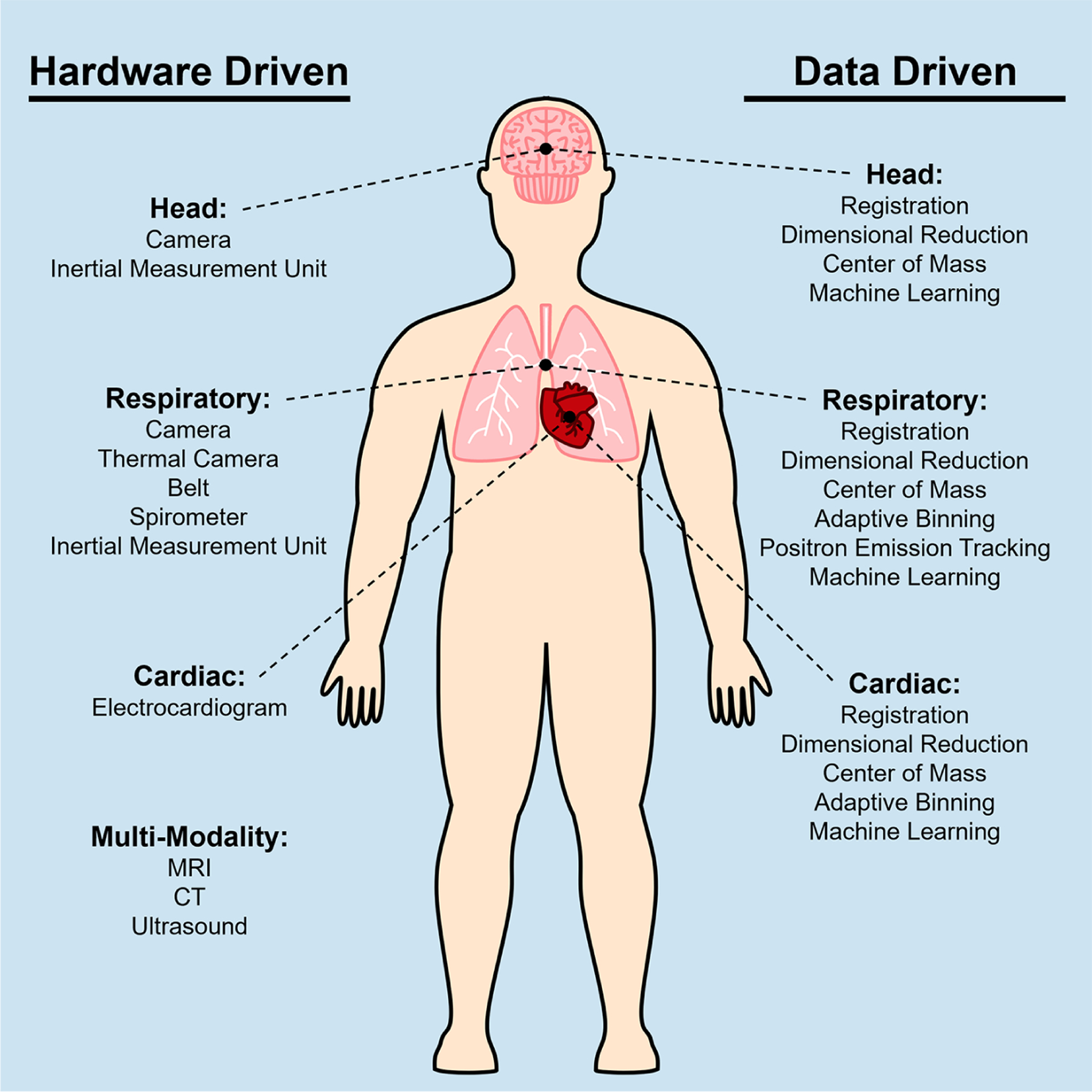
Diagram of techniques used to correct for head motion, respiratory motion, and cardiac motion for PET imaging. Multiple techniques have been developed based on the specific application when imaging certain anatomical areas, thus these techniques can be categorized based on the motion source that they are addressing. The left column shows a list of hardware driven techniques, and the right column shows data driven techniques.

Medical imaging often focuses on specific regions of the body. Common categorizations of general imaging targets are brain, head/neck, chest, abdomen, spine, upper limbs, and lower limbs. From each of these categories, PET imaging finds its use particularly in the head, chest, and abdomen to evaluate specific organs of interest such as the brain, lung, heart, and liver. Recent developments in PET imaging have enabled total body simultaneous imaging capability, making motion correction of the entire body critical. However, due to the different types of motion present in various regions of the body, motion needs to be specifically considered for each region, as depicted in Figure 1.

Subject motion can be categorized as voluntary or involuntary. Some common forms of voluntary motion are speaking and moving of the limbs due to patient non-compliance. Voluntary forms of motion are generally not an issue for PET imaging because they can typically be addressed by asking the subject to avoid movement. However, non-compliance (e.g., pediatric or dementia patients) or patients who experience severe discomfort can lead to voluntary motion. Involuntary motion sources are typically the main ones that are considered when designing motion correction strategies. The primary sources of motion that are taken into consideration in whole-body PET imaging are respiratory motion, cardiac motion, and bulk motion. While respiratory motion and cardiac motion only results from breathing and the beating of the heart, bulk motion encompasses any large-scale movements, such as moving of the arms or legs. When specifically imaging the chest or the abdomen, bowel activity and peristaltic motion are sometimes also considered as variables for motion correction. These motion sources can be further categorized as rigid or non-rigid motion. Rigid motion involves simple translation or rotation, hence head motion is commonly considered as rigid motion. On the other hand, non-rigid motion involves more complex motion that could include twisting or bending (5). As such, cardiac, respiratory, peristalsis, and bulk motion are all forms of non-rigid motion. From all these sources of motion, their impact varies depending on the body region.

The head serves as a crucial target for PET motion correction. The head, being the region of primary interest in many PET studies, is particularly susceptible to motion artifacts due to its anatomical complexity. Involuntary movements of the head can introduce unwanted artifacts, distortions, or inaccuracies in the resulting images. Motion correction methods aim to mitigate these issues by compensating for head motion and restoring the integrity of the acquired data. These methods employ sophisticated algorithms and techniques to estimate and correct the motion, ensuring that the resulting data or images accurately represent the intended information. By addressing head motion, motion correction methods enhance the quality and reliability of various applications, enabling more accurate analysis, diagnosis, and interpretation of the collected data. These head motion correction methods can be classified into categories such as image-based methods, sensory-based methods, retrospective methods, prospective methods, and hybrid methods. Chest and abdominal imaging are generally considered to have the highest need for motion correction (8); the presence of cardiac, respiratory, and peristatic motion can cause substantial displacement and deformation. Furthermore, bulk patient motion in combination with the previously mentioned motion sources span a variety of temporal patterns which makes image alignment and correction for this target anatomy challenging.

## 2 Hardware gating

Hardware-driven gating methods are commonly employed in PET imaging to address the challenges posed by respiratory motion. There are also other systems available to track other sources of motion or multiple sources of motion simultaneously. These hardware-based methods utilize external sensors or devices to track the patient’s respiratory cycle and synchronize the PET data acquisition accordingly. Several hardware-driven gating techniques have been developed to correct for motion artifacts in PET imaging. The majority of these techniques are applied as retrospective gating approaches.

### 2.1 Head motion

Due to the nature of head motion, there are limited hardware-based techniques that can be utilized. One option is the use of optical-based tracking systems. These systems use cameras to track head motion and observe mostly rigid motions of displacement and rotation. This is often achieved with markers being fixed to the subject and having the camera track the motion of these markers. Camera-based methods can also be achieved without the use of markers; they instead use facial features as markers for motion tracking (16). The use of this system is not limited to just correcting PET data but also sees use with other imaging modalities and multi-modality cases such as PET/MRI acquisitions. This technique has been demonstrated on Alzheimer’s disease patients and is able to improve the accuracy and reliability of PET/MR imaging for clinical populations (17). However, as a hardware-based system, the cameras used for motion tracking need to be set up prior to scanning and can be quite intrusive. The setup for the additional specialized equipment can increase scan time and introduce other errors stemming from the camera acquisition in the reconstructed images.

The rigid head motion can also be estimated using inertia measurement units (IMUs). Similar to applying a marker for tracking, IMUs can also be attached to subjects and be used to make motion measurements (18). An IMU is a sensor composed of an accelerometer and a gyroscope that makes real-time triaxial acceleration and velocity measurements. In some cases, a magnetometer is also included as a component to make magnetic field measurements and increase the degrees of freedom from six to nine. The use of this device was used in a study involving patients with cerebral palsy and found that the IMU was highly correlated with craniocervical movement for both healthy and cerebral palsy subjects (19). Although IMUs can be used for making motion measurements and has seen concurrent use for correcting cardiac and respiratory motion with PET imaging, few reports have utilized it concurrently with head imaging (20).

### 2.2 Cardiac motion

When it comes to chest and cardiac imaging, cardiac motion is the major motion source that needs to be addressed, in particular to ensure quantitative accuracy of tracer distribution (21). The conventional way to measure heart signal is through the use of a measured electrocardiogram (ECG) and then performing gating based upon that signal (22). Nonradiopaque electrodes are placed on the patient for continuous monitoring of ECG over the entire duration of a given scan protocol, the ECG signal then being stored serves as a basis for gating of associated scan data as it is acquired over time. ECG gating of cardiac image data allows accurate quantitative analysis of cardiac structure and function (23). The desire for a single static image with quantitative accuracy for analysis has resulted in several motion-correction methods being implemented. One such method is including left-ventricular (LV) segmentation plus motion vector tracking, which has demonstrated reduced myocardium thickness in static image reconstruction following adjustment of all cardiac phases to the end-diastolic motion state (24). Additionally, image registration between gated cardiac images has been explored; nonlinear registration has shown particularly promising results, resulting in a 46% reduction in noise in recent published work (21). Motion correction methods have been explored in greater depth when implemented during post-reconstruction processing, including the aforementioned works; however, implementation during the reconstruction process has shown promise in offering improved SNR and overall image quality, such as recent work using mass-preserving optical flow motion vectors calculated post-reconstruction and subsequently implemented into the reconstruction process (25, 26). The consideration of respiratory motion in cardiac imaging is also pertinent to image quality and has been implemented in dual-gated motion correction schemes (27).

### 2.3 Respiratory motion

Respiratory motion can introduce relatively large motions into PET imaging. Hence, many methods have been explored to address this motion source. To make respiratory motion measurements, a couple of different camera setups have been explored: marker tracking, depth profiling, and thermal imaging. The marker tracker is a simple setup involving a camera capturing the movements of a tracker, often reflective, fixed to a position on the subject. For depth profiling, two cameras are spatially calibrated for triangulation so that 3D profiling can be achieved (5, 28). Two optical cameras (Kinect, Microsoft Corporation, Redmond, Washington) were used in one study on ten patients and were able to achieve an average Pearson correlation coefficient of 0.74 between the measured abdominal signal and the data-driven signals (28). For thermal imaging, a thermal/infrared camera is used to capture patient breathing and used for breathing evaluation (29). Optical lasers have also been used as displacement sensors. Like optical cameras, this approach requires the subject’s abdomen to be exposed so that the respiratory motion can be captured (30). When comparing these methods with a respiratory belt, another hardware-based gating method, it has been shown that all these methods are well correlated and can generate motion or respiratory signals. One of the challenges with these methods, especially for thermal cameras, is the extensive processing of these images to make consistent and repeatable measurements. One commonly used form of an optical system for PET imaging are the Real-time Position Management (RPM) and Respiratory Gating for Scanners (RGSC) systems (Varian Medical Systems, Palo Alto, California). The RGSC system is able to provide more accurate 3D positions of markers with more robust spatial information compared to the 2D capabilities of RPM. The RGSC system uses a shielded camera, which helps eliminate background noise and stabilize the camera. Although the RGSC system proved to be as effective as RPM for patients with regular breathing patterns, or motion periods between 3 and 10 s, there are doubts surrounding its effectiveness with irregular breathing patterns. From testing the RGSC system, it achieved a 76% agreement within a ± 5% tolerance, matching the programmed “ground truth” data. In comparison, the RPM system had a 66% agreement within the ±5% tolerance, and the RGSC system had a 65% agreement when compared to the RPM measurements (31).

**FIGURE 2:**
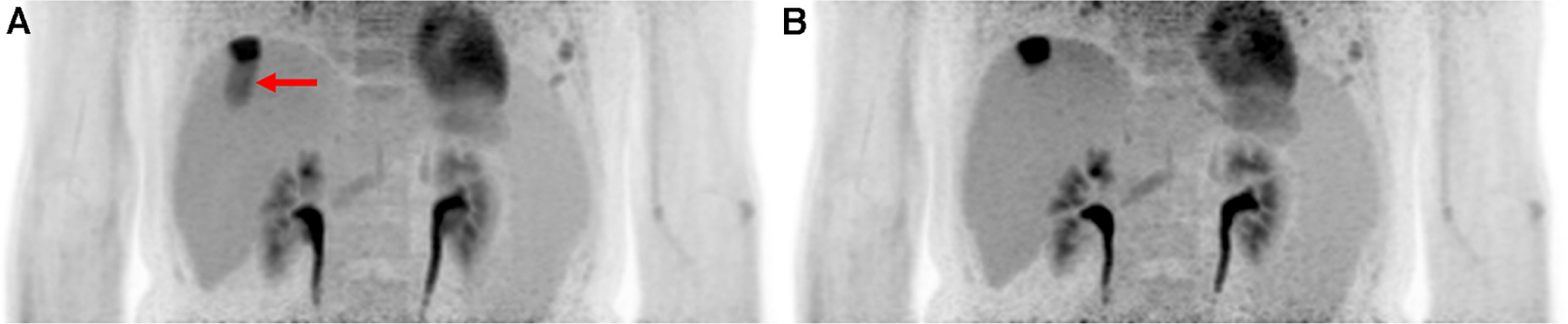
Implementing hardware-driven gating can provide drastic improvement to PET images by reducing motion artifacts. It helps improve SNR and remove motion artifacts. Abdominal PET images of the liver and kidneys are shown with (**A**) no gating and (**B**) respiratory belt gating. The red arrow shows a distinct motion artifact adjacent to an FDG-avid lesion in the ungated image.

A more common approach for measuring respiratory motion is the use of a respiratory belt (also known as a respiratory inductance plethysmography belt). This non-invasive device is placed around the patient’s chest or abdomen and measures the expansion and contraction of the thoracic or abdominal region during breathing. In some cases, this device is set up in a dual band mode for rib cage and abdominal signals. The strain gauge in this device can transmit real-time signals to the PET scanners and other imaging modalities, which will allow for the option for respiratory-triggered gating. This makes the respiratory belt a qualitative measure of the volume change correlated with breathing. This method has been accepted to be sufficiently accurate for estimating the respiratory rate. In one study the belt was reported to produce a signal reliability of 98.5%–98.8% (32). The simplicity of its setup and non-invasive nature makes this method popular clinically. However, its use is dependent on properly securing the belt in the correct position. Shallow breathing or improper placement can cause the belt’s accuracy to drop. An example of a respiratory gating using the respiratory belt can be seen in Figure 2.

The respiratory spirometric gating device is a more direct approach that operates by taking the breaths of the patient through a face mask to track the volume of air associated with inhalations and exhalations to determine the respiratory volume change. The spirometer can make high temporal resolution measurements and even operate effectively under abnormal breathing conditions. Although this device can provide accurate measurements, it does require some setup that can contribute to subject discomfort. Some spirometer setups utilize a mouthpiece instead of a face mask that forces breathing only through the mouth. Furthermore, an issue that can arise from this device is a drift of the volume baseline which can stem from flow measurement errors. Aside from this, it was found to be able to perform better than the RPM by being able to improve the detection of peak inhalation by 8% and reducing time lag by 260 ms (33).

IMUs have been applied to respiratory tracking but also have seen applications with dual gating cardiac and respiratory (34). A recently developed method using an inertial measurement unit based on microelectromechanical systems (MEMS) is used to make local measurements and estimate motion for different parts of the body. As it is a sensitive, accurate, and low-cost method, it can also make multiple measurements across the body. It was found to be able to achieve a mean absolute breathing error rate of 0.44 per minute and a low amplitude error of 0.24 cm (35). However, one of the inherent drawbacks of IMUs is the sensor’s susceptibility to accumulating errors over time.

Sensors of all sorts can be used to make motion measurements. Another strategy that is used to capture motion makes use of electromagnetic fields. For this system to work, a transmitter and receiver is necessary to observe the changes in the field due to motion. Since these types of sensors use electromagnetic fields, it has an advantage of being immune to occlusion but is susceptible to metallic objects that can distort electromagnetic fields. One implementation of this method makes use of continuous wave radar to produce 24 GHz electromagnetic waves. The 24 GHz frequency was chosen to allow for the EM waves to propagate through clothing, blankets, and other plastic coverings but will experience absorption and reflection once the wave reaches materials with high water content. These properties enable the use of continuous wave radar to track respiratory-related movements. Although it was unable to detect displacements smaller than 25*μ*m, the device was able to achieve high Pearson correlation coefficients when compared to a respiratory belt and a depth camera with values between 0.69 and 0.99 (36).

### 2.4 Multi-Modality approaches

Some acquisitions for PET are acquired in conjunction with other imaging modalities such as MRI, CT, or ultrasound. The use of multi-modality acquisition is rapidly increasing because other imaging modalities can provide more information at higher spatial and temporal resolutions that are not observed in PET. These other imaging modalities can also be used to extract motion information from multiple motion sources for motion correction in PET. As such, data-driven techniques are needed to analyze the other modalities’ image data to derive motion parameters or to generate a motion model.

MR-informed motion correction (MR navigation) is performed using a rigid-body co-registration of high framerate MRI such as echo planar imaging (EPI) that can be acquired periodically through a hybrid imaging protocol. The resulting motion parameters can then be used to correct motion in the PET data acquisition by realignment of PET frames. Motion estimates derived from the fMRI raw data were used to correct the PET data before image reconstruction using either a single-pass or two-pass reconstruction. The images blurring due to subject motion is reduced and the FDG uptake in the cortical region ribbon can be better appreciated qualitatively after motion correction. A mean relative change of ~4% in the regional SUVR was observed after motion correction even at the group level (37). Paired t-tests of the mean SUVR were performed showing that the measured difference was significant (37). The application of these methods is not limited to just human subjects but also find use in animal studies such as rabbits and primates. MRI data acquired is able to be used as a registration target to be incorporated into the PET list-mode reconstruction and correcting attenuation correction maps (38). MR navigators have varied approaches and successful commercial and research implementations have been studied in PET/MR (38).

A dual-gated approach was applied for PET using CT data to correct for respiratory and cardiac motion. The motion correction of the PET images was achieved by generating a model based on the CT data. This technique was ultimately able to reduce motion artifacts and increase SNR and CNR. SNR increased to 27.5 from 20.3 and CNR increased to 14.5 from 11.1 when compared to more traditional hardware-based dual-gating approaches. Although significance tests show no significant difference in the myocardium, significance was found in the blood pool (39). In another implementation, the size of hot spots, on average, decreased by 49.7% after applying the correction (40).

Ultrasound can also be used to capture motion data, specifically breathing motion. The displacement of internal organs can be captured using ultrasound as “organ configuration motion” (OCM) sensors. These sensors would have to be attached to the skin to generate motion-resolved images. This technique was tested on phantoms and two patients with small and large lesions. With these test cases, images were able to be generated using phase-based gating. Development on alternative strategies of applying the ultrasound data to improve the gating process could further improve the quality of the motion-corrected image (41).

### 2.5 Post-processing

The equipment mentioned earlier, which includes cameras, respiratory belt, spirometer, ECG, and IMU devices, provides the capability to track motion in real time. The signals generated by these tools can be utilized to prompt the collection of PET data at specific intervals within cycles. To decide which region of the acquired data is usable for image reconstruction, phase-based motion gating and amplitude-based motion gating are both post-processing techniques used to address the issue of respiratory or cardiac motion in PET data acquisition and reconstruction.

#### 2.5.1 Phase-based motion gating

**FIGURE 3:**
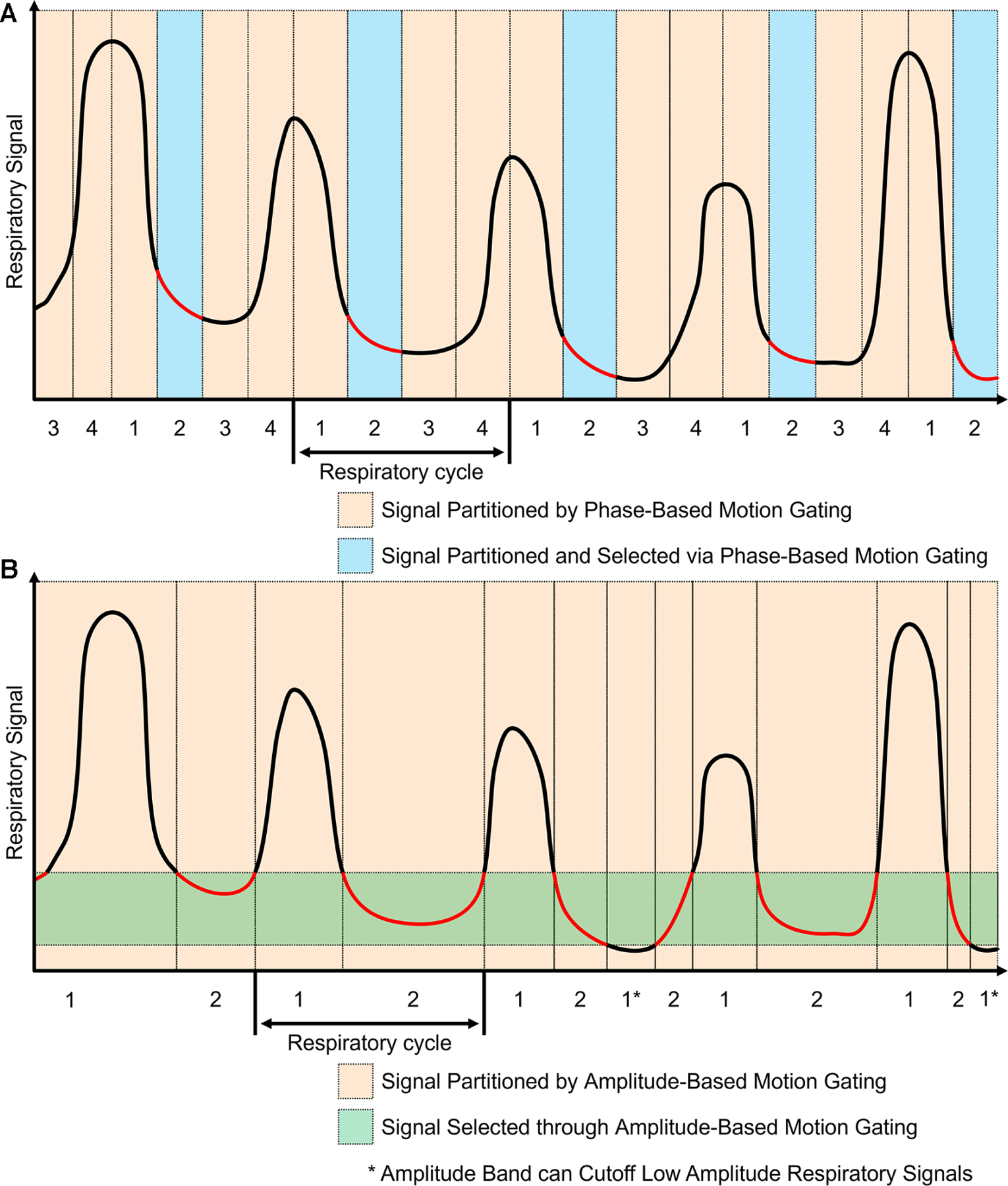
Phase-based and amplitude-based gating are two ways to process motion signals for motion gating. The red components represent regions used for image reconstruction. (**A**) Phase-based gating divides the motion signal into gates. Specific gates can be chosen for the corresponding PET data to be used for image reconstruction. Using this method, multiple images can be generated based on each phase gate. (**B**) Amplitude-based gating divides the motion signal based on the amplitude of motion. The PET data with motion that is within the specified amplitude band is kept for image reconstruction. Data with motion that is outside of the amplitude band is discarded.

The workflow for phase-based motion gating (PBG) typically starts with defining a periodic motion cycle, which could be a cardiac or respiratory cycle, depending on the organ of interest. Subsequently, a physiological signal that mirrors this motion is recorded and synchronized with the imaging data. Following synchronization, the motion cycle is divided into several distinct phases, and the imaging data is sorted accordingly into these phases, with each phase representing a unique gate. An example of this phase-based gating process can be seen in Figure 3A where the motion signals are partitioned into sections and only the regions with the red line are kept for image reconstruction. In the PBG approach, not all gates are considered equally suitable. Some may not accurately represent the phase of the physiological motion being studied and are therefore discarded. Only those gates that meet specific criteria and accurately represent the phase of motion are retained for image reconstruction (42). The choice of appropriate phases or gates in PBG is a critical aspect and is heavily influenced by various factors. For instance, in respiratory motion gating, a phase where the patient’s breathing is relatively stable will be preferred to minimize motion blur. This phase often corresponds to the end of exhalation, a point where breathing is typically most stable. Using data from this phase can provide clear, high-quality images with reduced motion artifacts.

#### 2.5.2 Amplitude-based motion gating

Amplitude-based motion gating (ABG) is a specialized technique utilized in PET/CT imaging to account for the variability introduced by respiratory motion. In contrast to phase-based gating, which segments the motion cycle into time-based intervals, ABG considers the displacement or amplitude of the physiological motion itself (42). This approach entails identifying a specific segment within the amplitude range of the respiratory signal to be used for image reconstruction. Rather than encompassing the entire breathing cycle, this selection often targets a narrow range that corresponds to a significant phase of respiration, such as the lung’s contraction phase. By concentrating on a specific state, ABG aims to maintain consistency in the shape and position of the imaged organs throughout the scanning process. This method helps minimize motion-induced artifacts in the resulting scans, thereby enhancing image clarity and accuracy. This effect can be seen in Figure 3B where an amplitude band was selected to keep most of the end of exhalation; some components that went below the amplitude band were discarded. Phase-based motion gating would be a more appropriate choice for cardiac imaging or cyclic respiratory movements (43, 44). This is because, in such instances, the movement is patterned cyclically. Therefore, a specific segment of the cycle consistently represents the same phase of the heartbeat or respiratory cycle, which results in a stable shape of the organs involved. On the other hand, amplitude-based gating may be better suited for imaging when the motion is less predictable, especially in situations with varying amplitude or a changing baseline from multiple motion sources (43, 44). Under these circumstances, ABG often outperforms PBG, providing more accurate results due to its ability to better handle these irregularities.

## 3 Data-driven gating

**FIGURE 4:**
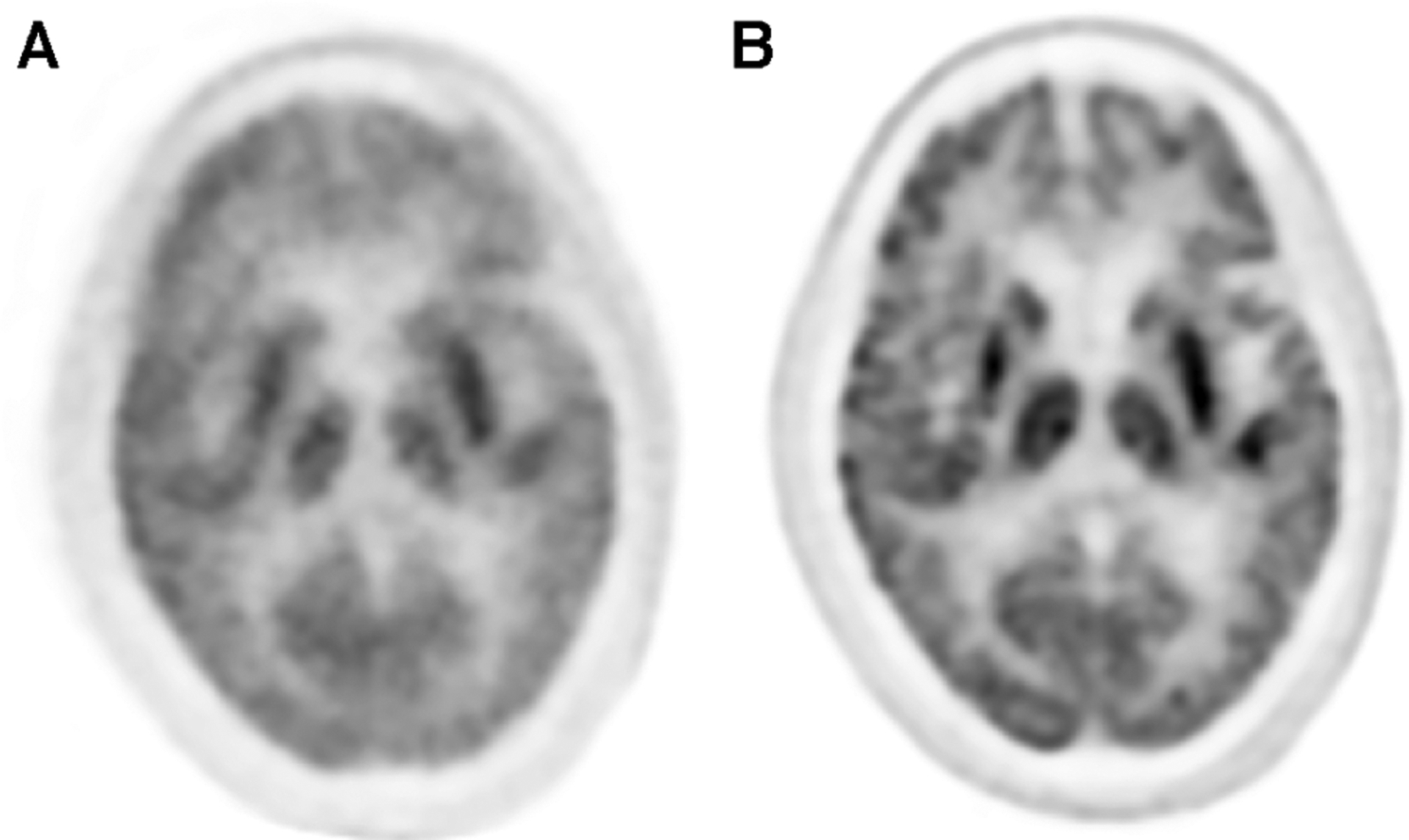
The application of data-driven gating can vastly improve image quality of PET images. (**A**) Uncorrected images can show lots of blurring and other motion artifacts. (**B**) Data-driven motion correction using an image registration approach was applied to this brain image and shows much greater clarity than the uncorrected version.

Unlike hardware-driven gating, data-driven gating methods utilize the PET data itself to retrospectively identify motion and make image corrections accordingly. These techniques involve analyzing the acquired PET data to extract motion information, such as using motion estimation algorithms or registration algorithms to align the dynamic PET frames. By retrospectively identifying and correcting for motion in the data, data-driven gating techniques enable improved image quality and quantitative accuracy in PET imaging, offering an additional approach that can be used with hardware-driven gating approaches to mitigate the effects of motion artifacts. An example of the impact that data-driven gating can have on brain images can be seen in Figure 4.

### 3.1 Head motion

One data-driven method used to address head motion is 3D center of mass (COM), which is a technique that can automatically detect head motion during PET scans without the need for external tracking devices. This algorithm uses all three dimensions of the center of tracer distribution trace and self-adaptive to noise levels. COM is shown to be effective in detecting radical translation motion errors in slowly varying tracer distribution caused by the motion tracking hardware and can be used to compare different motion estimation methods (45). Also, from studies it was found that the results of this method are very comparable to belt-based gating. The results found from both methods were highly correlated, with 0.99 in phantom and 0.94–0.97 in clinical patients (45). Similar results were also found when compared with optical hardware-based motion tracking. SUV differences were found to be 1.0%±3.2% across 290 subjects (23 datasets) in gray matter regions (46). Testing on simulated and real human datasets showed that 3D COM outperformed other motion correction methods, including one-direction COM and frame-based image registration, and yielded comparable results to gold standard hardware-based motion tracking method. The algorithm has the potential to improve the image quality and accuracy of PET scans, particularly for clinical populations where head motion can be a significant challenge, but further validation in larger and more diverse patient populations is needed. This proposed motion correction method yielded −0.3±2.8% and −0.4±3.2% brain region error for ^18^F-FDG and ^11^C-RAC, respectively, across 10 subjects with larger head motions for each tracer (47).

Registration is also a simple approach that can be used to estimate motion. Prior to applying registration, PET images undergo ultrafast reconstruction using very short frames that are automatically set and adjusted at each frame to ensure consistent and scattering events pre-frame were performed for the entire scan duration. Following this, image-based registration is performed on these frames to estimate motion. Rigid registration was performed using least-square metric and gradient descent optimization. The 6 degrees of freedom of motion were estimated, with an accuracy of less than 1 mm. The reference non-AC emission frame for image registration is selected for alignment between PET reconstruction and the attenuation map (3). The maximum difference in SUV max of the parietal lobe between motion-corrected and non-motion-corrected reconstructions is 1.5%±2.7%, with a maximum discrepancy of 6.6% (48). To obtain high enough SNR, data frames are often sampled at lower temporal resolutions, but it has been found that very short frames of less than 1s can be used to provide accurate and quick motion estimates (49). Alternatively, a data-driven approach utilizing Time-of-Flight (TOF) weighted positron emission particle tracking algorithms enables fully automated head motion detection. The TOF-PET algorithm has been validated to detect motion using a 500 ms window. The use of event-based correction yields motion artifact-free images. A frame-by-frame image registration-based gold standard, created for this study, and the automated Line of Response (LOR)-based correction demonstrate similar results, with Jaccard similarity indices in the range of 92.5%±4.8% for the former and 93.2% ±4.5% for the latter (6). The Jaccard similarity index is defined as the intersection over the union between transformed images and reference images.

Dimensional reduction, specifically principal component analysis (PCA), has seen use as a method for making head motion correction. PCA can decompose PET data into its principal components to isolate head motion. This method was validated using phantom and patient acquisitions and were found to be effective in identifying motion occurrences and producing motion-free images with increased sharpness compared to fixed-framed approaches. Image sharpness of the non-corrected images ranged from 79%–82% of the motion-free image sharpness and PCA frames increased sharpness to 97.9% for both acquisition and containing movement. This technique not only increases the average image sharpness by the same amount as the fixed frame approach, but it reduced the number of reconstruction and registrations by a factor of 3.4 on average. The proposed method offers the advantage of retrospective motion estimation and potentially reduces motion for long PET frames, improving the accuracy and reliability of PET imaging for brain disorders (50).

Modeling motion is a strategy that is also used to characterize motion which can be implemented into kinetic modeling. This direct reconstruction approach utilizes an expectation maximization algorithm that assumes emission data is Poisson distributed. This implementation was able to decrease the coefficient of variation in a simulated set by 35%–48%, in a [^11^C] AFM set by 39%–43%, and in a [^11^C]UCB-J set by 30%–36% (51). To achieve motion gating, deep learning models can also be used for correcting head motion. One example of a deep learning model for head motion correction is the DL-HMC methodology that consists of three components: (i) PET input data encoder layers, (ii) regression layers to estimate the six rigid motion transformation parameters, and (iii) feature-wise transformation (FWT) layers to condition the network to tracer time-activity. The input of DL-HMC is sampled pairs of one-second 3D cloud representations of the PET data and the output is the prediction of six rigid transformation motion parameters. The network was trained in a supervised manner using optical motion tracking information as gold standard. The algorithm quantitatively evaluated DL-HMC by comparing to gold-standard optical measurements and qualitatively evaluated the reconstructed images as well as performed region of interest standard uptake value (SUV) measurements. The network was trained by minimizing the network’s mean square error (MSE) between the predicted motion estimate θ and optical reference θ using Adam optimization with initial learning rate 5e-4, *γ*=0.98, and exponential decay with step size 200. Because of GPU and CPU memory constraints, a smart caching dataset was used to replace 25% of the data (1,024 samples) for each epoch with new samples (52).

Another developed motion-correction approach is aided by conditional generative adversarial network (cGAN) methodology that allows reliable, data-driven determination of involuntary subject motion during dynamic ^18^F-FDG brain studies. The GAN was trained using 70% of the total datasets, which were corrected for motion using MR navigators. The estimated motion parameters were then used to extract the Image Delivered Input Function (IDIF) from the motion-corrected dynamic sequence. The resulting cGAN mappings were then applied to the test datasets, producing artificially generated low-noise images from early high-noise PET frames. These low-noise images were then co-registered to the reference frame, yielding 3-dimensional motion vectors (53).

### 3.2 Cardiac and respiratory motion

**FIGURE 5:**
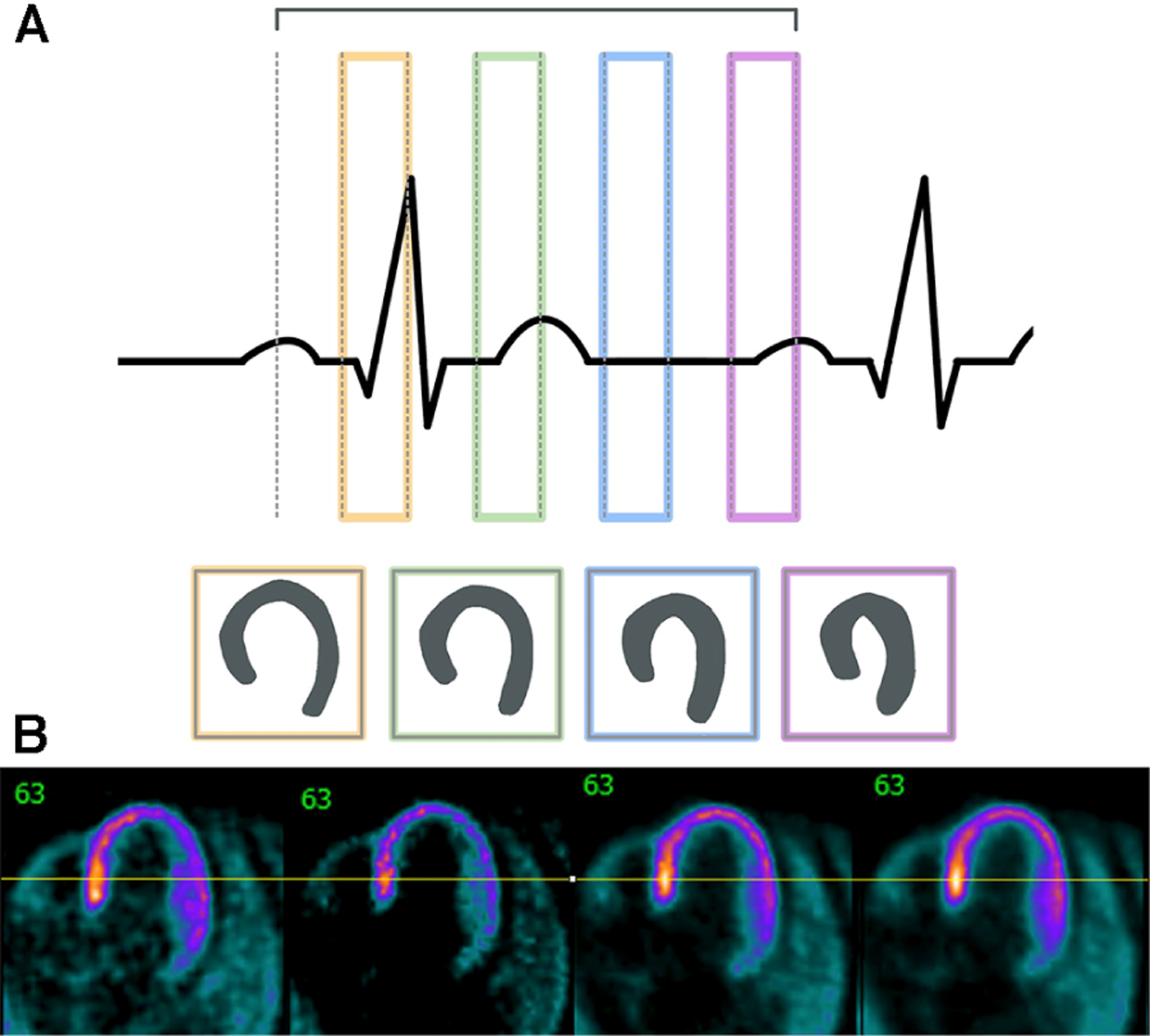
Cardiac gating must take into account the different phases of the cardiac cycle. (**A**) This outline shows the different sections of the cardiac cycle with the PQRST peaks and how different stages could affect cardiac outlines. (**B**) Sample images shown here are non-gated, EKG-gated, cardiac-gated, and dual-gated from left to right.

Due to the close proximity of cardiac and respiratory motion, especially when performing chest or abdominal imaging, many data-driven gating approaches are implemented as either dual-gating approaches that account for both motion sources or as respiratory-only approaches. An example of the impact that dual gating can have on cardiac images can be seen in Figure 5.

One technique that is very similar to hardware-driven techniques is the positron emission tracking (PeTrack) method. PeTrack operates by tracking a positron emission source as a marker and uses the motion of this marker to generate respiratory motion signals from dynamic 4D PET data. PeTrack demonstrates potential in situations where there are irregular breathing patterns and provides a relatively simply patient setup process. Despite this, PeTrack still has challenges, such as the potential interference from high count rates of radiopharmaceutical tracers and additional radiation exposure. Further studies are needed to optimize its performance under various imaging conditions (54).

Dimensional reduction is a data-driven gating strategy that is often used for addressing cardiac and respiratory motion correction. Two main approaches that fall under this category are principal component analysis (PCA) and Laplacian eigenmaps. Both methods can be used to identify the dominant patterns of variability associated with motion in a dynamic 4D PET dataset. By decomposing the PET data into its principal components, the data can be classified into different motion bins and reconstructed as motion-corrected images based on the identified patterns. The distinguishing difference between PCA and Laplacian eigenmaps is that PCA operates on a linear basis while Laplacian operates as a nonlinear manifold learning method. By applying PCA for motion correction, one study was able to improve lesion SUV max values from 7.9 to 9.0 (55). By combining the sensitivity profile with PCA and Laplacian eigenmaps, another study was able to increase correlation with MR data to from 0.58 to 0.74 using PCA and from 0.42 to 0.7 using Laplacian eigenmaps respectively (56).

An alternative approach in data-driven gating is to evaluate the counts acquired in the raw PET data. Hence this process would occur before the reconstruction step and look for changes associated with motion in raw PET data. Motion would create variations in the PET data that can be correlated with external motion signals. Assuming motion is consistent and its fluctuations are mostly comprised in a range of frequencies, gating can be performed based on the periodicity associated with the frequency of motion. Applying a frequency analysis for gating resulted in an increase of the SUV max in lesions by 28.6% compared to an ungated approach (57). A more advanced approach to this method is to apply multi-binning or adaptive binning. Multi-binning performs gating on the PET data into different phases which would result in multiple images that can be reconstructed. This would allow for further evaluation and comparison of the images to determine which is the best. The performance of the multi-bin respiratory gating was found to be more effective in motion correction than applying end-respiratory gating (58). The adaptive binning method decides which components are to be gated out based on the local motion amplitude while no gating is applied in static regions. The application of this approach was able to reduce noise when compared to conventional gating reconstructions and make a better trade-off between resolution and noise (59). An inherent issue with gating is that it would throw away data that is not in the proper bins, which would increase the noise in the reconstructed image.

COM techniques can be used to calculate the position of the center of mass of the tracer uptake distribution within the acquired PET data at different respiratory phases. By tracking the movement of the COM, the respiratory motion can be estimated and used to gate the PET data during image reconstruction. The COM method in one study was able to achieve a correlation of 0.85 with device-based signals (60). Compared specifically with respiratory belts, correlation coefficients were found ranging from 63% to 89% (61). Based on the gated images, this data-driven approach was able to improve lesion contrast compared to the uncorrected image and produced comparable quality to the device-based corrected images. In an alternative implementation where COM was combined with a frequency filter, comparisons with simulations showed that there was a higher probability that the addition of this filter was able to improve the COM method and increase the probability that the voxel values are accurate (62).

Another commonly used technique to address respiratory and cardiac motion is registration. Registration is utilized to generate a set of reconstructed images at different motion states to estimate the motion experienced. Various methods for image registration and implementation have been published, falling into two broad types of registration: rigid and nonrigid (63). Rigid motion registration consists of generating some non-anatomy-deforming transformation for a given motion state relative to a selected target motion state. This transformation accounts for translational and rotational motion in the X, Y, and Z directions, and is considered suitable for motion correction of brain PET images (63, 64), but it is not likely to be sufficient for other regions of the body. Nonrigid registration produces a deforming or anatomy-warping transformation map for each image motion state in relation to the chosen target motion state (64). Also known as elastic image registration, this general category of registration algorithm is considered suitable for and has been applied in image data with cardiac and respiratory motion present (64). One example of an elastic algorithm implemented commercially is Q.Freeze (GE Healthcare, Chicago, Illinois) which reconstructs multi-bin respiratory gating images, followed by nonrigid registration, and averaging into a single volume (65). Image registration for motion correction can be implemented either during the image reconstruction process or after reconstructing a set of images at different motion states. The latter approach involves registration and the application of transformation maps to combine the images into a single static motion-corrected image. An example of an implementation of the former approach is OncoFreeze (Siemens Healthcare, Erlangen, Germany) which derives a blurring kernel from sub-images to be employed during the image reconstruction process to generate a motion-corrected image (66).

In recent years, machine learning models and networks have emerged as powerful tools for data-driven gating in PET imaging. These models can learn complex motion patterns from the acquired PET data and predict motion information that is then used to guide the image reconstruction process. Machine learning-based data-driven gating approaches have shown promising results in improving PET image quality and accuracy. It was found from one deep learning model that the normalized root-mean-square error of the deep learning model, iterative registration, and ungated methods was 24.3%, 31.1%, and 41.9%, respectively (67). This shows that this particular deep learning model was able to further improve the accuracy of the resulting image beyond that achieved from iterative registration.

## 4 Applications and challenges in parametric dynamic whole-body PET

While the methods previously mentioned have been shown to work in conventional PET imaging, the applications of these methods are not trivial when it comes to implementing them in dynamic whole-body PET imaging. Dynamic whole-body PET acquisitions can be divided into two categories: short axial FOV and long axial FOV. Dynamic acquisitions on short axial FOV PET systems have previously been confined to single bed protocols, but recent clinical implementations have been using multi-bed protocols (68, 69). Whole-body PET imaging is more susceptible to motion artifacts from various sources due to extended imaging coverage. With longer axial coverage from a whole-body (or nearly whole-body) PET acquisition, images can simultaneously include multiple regions of interest and would provide comprehensive coverage with substantially higher temporal resolution. However, the inherent drawback from a longer axial acquisition would be multiple motion sources that have unique impacts in different regions of the body. Periodic patient movements in each PET bed during these acquisitions can be mitigated with gating but would have trouble with irregular body motions, leading to smearing and partial volume effects (68, 70). A mixed approach using motion-tracking hardware or other imaging modalities along with simple data-driven techniques such as registration can be used to address motion. This process can improve the precise alignment of the data acquired and thereby significantly improving the delineation of ROIs, leading to more accurate and accurate kinetic parameters (71). The Patlak correlation-coefficient could be used for voxel-wise thresholding to filter out motion-corrupted data during parameter estimation (69).

At the time of publication of this review, few studies have studied long axial FOV whole-body in PET imaging due to conventional PET scanners requiring a multi-bed protocol and therefore not able to acquire truly simultaneous whole-body data (72). Simultaneous PET/MRI acquisitions are also not feasible since current systems do not have a long enough axial coverage and would also require a multi-bed protocol. Motion tracking that has previously locally worked on brain and respiratory motion may struggle with complex whole-body motion. However, without adequately accounting for motion, significant errors could arise in critical kinetic quantifications (i.e., SUV) essential for parametric PET analysis and kinetic modeling, especially for prolonged acquisitions longer than 60 min (73). Therefore, body-region specific reconstruction with motion-correction may be necessary, even in whole body acquisitions. The primary approaches are largely data-driven techniques as detailed herein. Image-based registration approaches have also found success, improving SUVmean values up to 12.89% (72). While motion tracking hardware and data-driven methods have been able to correct for motion, deep learning approaches have been increasingly showing an advantage in applying a non-rigid correction that can improve kinetic modeling in total-body dynamic PET imaging (74).

## 5 Discussion

**FIGURE 6:**
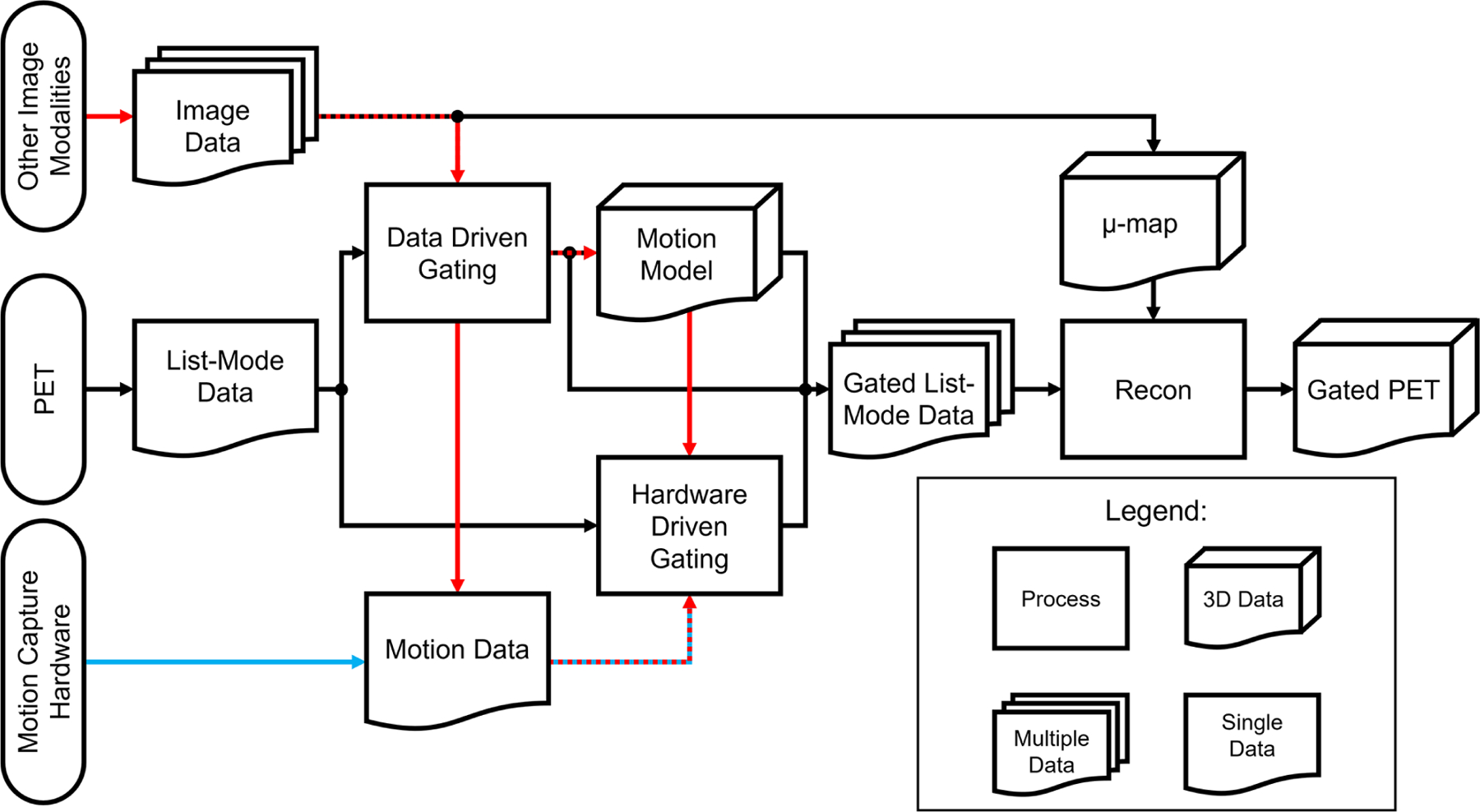
Processing flowchart for hardware-driven and data-driven gating. The red pathway represents processing pipelines of motion gating stemming from multi-modality PET imaging which can include images or data acquired from other sources such as MRI, CT, or ultrasound. The blue pathway represents the general processing pipeline for hardware-driven gating.

The choice between hardware-driven and data-driven gating techniques in PET imaging is an important consideration in addressing motion artifacts. Both approaches have their advantages and limitations, and selecting the most appropriate method depends on several factors, including the specific imaging setup, available resources, biological region of interest, and the expected motion information that will be captured. Choosing different methods can result in different processes needed to analyze the acquired data. A simplified outline of this process can be seen in the flowchart in Figure 6. From Figure 1, a categorization is provided of some of the many methods that have been explored in this paper and includes the biological regions in which these methods could be used for motion gating. Although many methods listed in Figure 1 show promise in addressing motion, their performance could change as PET scanners increase in capability and resolution. As such, future PET scanners would require re-evaluations of these techniques to determine any changes in efficacy. Comparisons between each method can be difficult due to the multitude of available methods and the metrics used to evaluate success. Often, statistics used to evaluate these gating methods are Pearson correlation coefficient, standard uptake value (SUV), signal-to-noise ratio (SNR), contrast-to-noise ratio (CNR), target volume change, image clarity score, structural similarity index measure (SSIM), mean squared error (MSE), and mean absolute error (MAE). The expectation from applying these motion gating schemes is a decrease of motion artifacts which would lead to apparent lesion volumes (target volumes) to decrease. As such, there would be an increase in image clarity, SNR/CNR, and accuracy of SUV. Under the scope of oncology, it is expected that the application of these methods would increase the SUV in lesions, which tend to be blurred or smeared as a result of motion. Ultimately, the application of these methods is expected to decrease the error statistics derived from MSE or MAE.

Hardware-driven gating methods, such as phase-based gating and amplitude-based gating, utilize external sensors or devices to track patient motion and synchronize PET data acquisition accordingly. These techniques have the advantage of real-time motion monitoring, providing immediate feedback on the patient’s respiratory or cardiac cycle. Hardware-driven gating is particularly effective in scenarios where precise real-time motion tracking is critical, such as in dynamic or time-sensitive imaging protocols. It allows for accurate gating based on external measurements, reducing the impact of motion artifacts.

On the other hand, data-driven gating techniques, including motion estimation algorithms and registration algorithms, rely on the PET data itself to retrospectively identify and correct for motion. These techniques analyze the acquired PET data to extract motion information, enabling retrospective gating based on the inherent motion patterns within the data. Data-driven gating has the advantage of not requiring additional hardware or sensors, making it more cost-effective and versatile. It can also provide more comprehensive motion information by accounting for multiple sources of motion such as respiratory and cardiac motion captured within the PET data. Data-driven gating is particularly beneficial when external motion tracking devices are not available or when more detailed motion analysis is desired.

However, it is important to acknowledge the limitations of each approach. Hardware-driven gating techniques rely on the accuracy and reliability of the external sensors or devices used for motion tracking. Factors such as patient compliance, shallow breathing, or irregular motion patterns can introduce errors or limitations in hardware-driven gating. Furthermore, hardware-driven gating may not capture all sources of motion, such as internal organ motion or bulk motion, which can still impact image quality. The number of hardware-based techniques are also all localized based on the region of interest and are not able to address bulk motion.

Data-driven gating techniques depend on the quality and fidelity of the acquired PET data. The accuracy of motion estimation algorithms and registration techniques can be influenced by factors such as image noise, tracer uptake variations, or the presence of motion artifacts themselves. One challenge for whole body PET imaging is that the field of view includes multiple organs and therefore multiple motion sources. This means that algorithms developed for specific organs or applications may not have a trivial adaptation to whole-body imaging. Careful optimization and validation of data-driven gating algorithms are essential to ensure accurate motion estimation and reliable motion correction. If the patient’s motion consists of gradual drifts or frequent and rapid displacements, external motion tracking methods are generally more suitable since they offer a higher sampling frequency (>30 Hz) and better spatial sensitivity (<1 mm). The development of long axial field of view PET scanners with extremely high sensitivity relative to conventional scanners will only make data-driven gating approaches more viable.

The future of motion correction strategies in PET imaging is increasingly promising. With continuous advancements in hardware- and data-driven gating techniques, the field is observing novel methods being developed that can provide more options with higher accuracy to address specific motion sources. Further developments in this field, including the utilization of inertial measurement units and the optimization of motion modeling through machine learning and deep learning, are poised for improvements in accuracy, efficiency, and practicality. These improvements will ultimately pave the way for enhancements in image quality, diagnostic precision, and quantitative measurements.
